# *cis*-Bis(acetonitrile-κ*N*)bis­(2,2′-bipyridine-κ^2^ *N*,*N*′)ruthenium(II) tetra­fluoridoborate

**DOI:** 10.1107/S1600536811053864

**Published:** 2011-12-21

**Authors:** Ying Wang, Feng Xu, Wei Huang

**Affiliations:** a Department of Chemistry and Chemical Engineering, Lianyungang Teachers College, Lianyungang 222006, People’s Republic of China; b State Key Laboratory of Coordination Chemistry, Nanjing National Laboratory of Microstructures, School of Chemistry and Chemical Engineering, Nanjing University, Nanjing 210093, People’s Republic of China

## Abstract

In the cation of the title compound, [Ru(CH_3_CN)_2_(C_10_H_8_N_2_)_2_](BF_4_)_2_, the Ru^II^ atom is six-coordinated in a distorted octa­hedral geometry by the N atoms of the two 2,2′-bipyridine (bpy) ligands and two *cis*-arranged acetonitrile mol­ecules. The dihedral angles formed by the pyridine rings of the bpy ligands are 8.86 (12) and 10.12 (14)°. In the crystal, the cations and anions are linked by C—H⋯F hydrogen bonds into a three-dimensional network.

## Related literature

For the structures of related complexes, see: Chattopadhyay *et al.* (2004 ▶); Cordes *et al.* (1992 ▶); Heeg *et al.* (1985 ▶); Xu & Huang (2007 ▶).
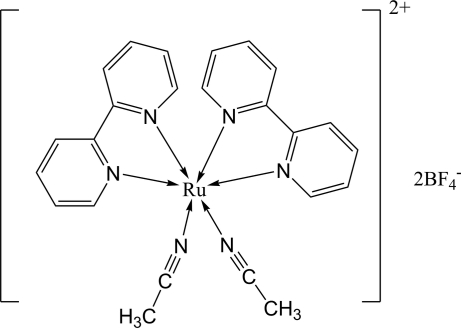


## Experimental

### 

#### Crystal data

[Ru(C_2_H_3_N)_2_(C_10_H_8_N_2_)_2_](BF_4_)_2_
*M* *_r_* = 669.17
Monoclinic, 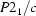

*a* = 10.5648 (7) Å
*b* = 24.0246 (17) Å
*c* = 10.4561 (7) Å
β = 90.253 (1)°
*V* = 2653.9 (3) Å^3^
*Z* = 4
Mo *K*α radiation
μ = 0.67 mm^−1^
*T* = 291 K
0.16 × 0.14 × 0.12 mm

#### Data collection

Bruker SMART CCD area-detector diffractometer
Absorption correction: multi-scan (*SADABS*; Bruker, 2000 ▶) *T*_min_ = 0.900, *T*_max_ = 0.924
13281 measured reflections
4680 independent reflections
3326 reflections with *I* > 2σ(*I*)
*R*_int_ = 0.045

#### Refinement

*R*[*F* ^2^ > 2σ(*F* ^2^)] = 0.046
*wR*(*F* ^2^) = 0.121
*S* = 0.95
4680 reflections
372 parameters
H-atom parameters constrained
Δρ_max_ = 0.76 e Å^−3^
Δρ_min_ = −0.41 e Å^−3^

### 

Data collection: *SMART* (Bruker, 2000 ▶); cell refinement: *SAINT* (Bruker, 2000 ▶); data reduction: *SAINT*; program(s) used to solve structure: *SHELXTL* (Sheldrick, 2008 ▶); program(s) used to refine structure: *SHELXTL*; molecular graphics: *SHELXTL*; software used to prepare material for publication: *SHELXTL*.

## Supplementary Material

Crystal structure: contains datablock(s) global, I. DOI: 10.1107/S1600536811053864/rz2684sup1.cif

Structure factors: contains datablock(s) I. DOI: 10.1107/S1600536811053864/rz2684Isup2.hkl

Additional supplementary materials:  crystallographic information; 3D view; checkCIF report

## Figures and Tables

**Table 1:** Hydrogen-bond geometry (Å, °)

*D*—H⋯*A*	*D*—H	H⋯*A*	*D*⋯*A*	*D*—H⋯*A*
C2—H2⋯F1^i^	0.93	2.50	3.179 (7)	130
C7—H7⋯F6^ii^	0.93	2.47	3.373 (7)	165
C9—H9⋯F4^iii^	0.93	2.42	3.299 (7)	158
C12—H12⋯F8	0.93	2.54	3.459 (7)	167
C14—H14⋯F2^iv^	0.93	2.33	3.238 (8)	164

Symmetry codes: (i) 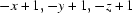; (ii) 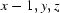; (iii) 
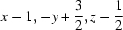
; (iv) 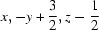.

**Fig. 1.**
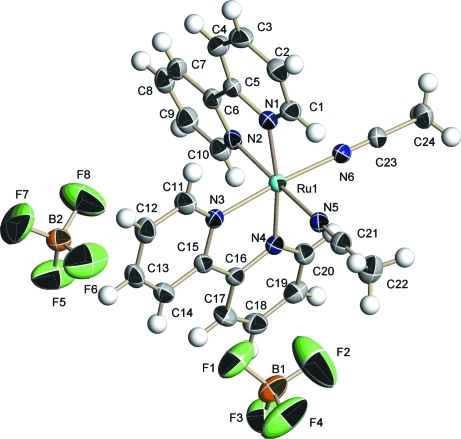
ORTEP drawing of the title compound with displacement ellipsoids drawn at the 30% probability level.

## supplementary crystallographic information

### Comment

The structures of *cis*-bis(acetonitrile)bis(2,2'-bipyridine) ruthenium(II) diperchlorate (Chattopadhyay *et al.*, 2004), *trans*-bis(acetonitrile)bis(2,2'-bipyridine) ruthenium(II) diperchlorate (Cordes *et al.*, 1992), and *cis*-bis(acetonitrile)bis(2,2'-bipyridine)ruthenium(II) hexafluorophosphate (Heeg *et al.*, 1985; Xu & Huang, 2007) have been reported previously. We present herein the crystal structure of the title compound (I) with the tetrafluoroborate counterions.

The atom-numbering scheme adopted for the title compound is shown in Fig. 1. The ruthenium(II) ion is six-coordinated in a distorted octahedral geometry by the nitrogen atoms form two 2,2'-bipyridine and two *cis*-arranged acetonitrile molecules. The six Ru—N bond lengths are in the range from 2.042 (4) to 2.060 (4) Å, and are comparable with those reported in the literature. The presence of coordinated acetonitrile molecules and free tetrafluoroborate counterions is confirmed by the characteristic absorptions of its FT–IR spectrum. The N1/C1-C5—N2/C6-C10 and N3/C11-C13—N4/C16-C20 pyridine rings within the 2,2'-bipyridine ligands are tilted by 8.86 (12) and 10.12 (14)°, respectively. In the crystal structure cations and anions are linked by C—H···F hydrogen bonds (Table 1) into a three-dimensional network.

### Experimental

The title compound was prepared by our previously reported method (Xu & Huang, 2007) except that sodium tetrafluoroborate was used. Single crystals suitable for X-ray diffraction measurement were obtained after 5 days on slow evaporation of an acetonitrile solution at room temperature. Elemental analysis: calculated for C_24_H_22_RuN_6_B_2_F_8_: C 43.08, H 3.31, N 12.56%; found: C 43.29, H 3.62, N 12.34%. Main FT–IR absorptions (KBr plates, cm^-1^): 3003 (w), 2293 (*m*), 2252 (*s*), 1606 (*m*), 1462 (*s*), 1421 (*s*), 1084 (*versus*), 1038 (*versus*), 918 (*m*), 764 (w) and 752 (w).

### Refinement

The non-hydrogen atoms were refined anisotropically, whereas the H atoms were placed in geometrically idealized positions (C—H = 0.93–0.96 Å) and refined as riding atoms, with *U*_iso_(H) = 1.2*U*_eq_(C) or 1.5*U*_eq_(C) for methyl H atoms.

### Crystal data

**Table:**

[Ru(C_2_H_3_N)_2_(C_10_H_8_N_2_)_2_](BF_4_)_2_	*F*(000) = 1336
*M**_r_* = 669.17	*D*_x_ = 1.675 Mg m^−^^3^
Monoclinic, *P*2_1_/*c*	Mo *K*α radiation, λ = 0.71073 Å
Hall symbol: -P 2ybc	Cell parameters from 3573 reflections
*a* = 10.5648 (7) Å	θ = 2.6–24.0°
*b* = 24.0246 (17) Å	µ = 0.67 mm^−^^1^
*c* = 10.4561 (7) Å	*T* = 291 K
β = 90.253 (1)°	Block, red
*V* = 2653.9 (3) Å^3^	0.16 × 0.14 × 0.12 mm
*Z* = 4	

### Data collection

**Table:**

Bruker SMART CCD area-detector diffractometer	4680 independent reflections
Radiation source: fine-focus sealed tube	3326 reflections with *I* > 2σ(*I*)
graphite	*R*_int_ = 0.045
φ and ω scans	θ_max_ = 25.0°, θ_min_ = 1.9°
Absorption correction: multi-scan (*SADABS*; Bruker, 2000)	*h* = −12→12
*T*_min_ = 0.900, *T*_max_ = 0.924	*k* = −15→28
13281 measured reflections	*l* = −12→12

### Refinement

**Table:**

Refinement on *F*^2^	Primary atom site location: structure-invariant direct methods
Least-squares matrix: full	Secondary atom site location: difference Fourier map
*R*[*F*^2^ > 2σ(*F*^2^)] = 0.046	Hydrogen site location: inferred from neighbouring sites
*wR*(*F*^2^) = 0.121	H-atom parameters constrained
*S* = 0.95	*w* = 1/[σ^2^(*F*_o_^2^) + (0.0705*P*)^2^] where *P* = (*F*_o_^2^ + 2*F*_c_^2^)/3
4680 reflections	(Δ/σ)_max_ = 0.001
372 parameters	Δρ_max_ = 0.76 e Å^−^^3^
0 restraints	Δρ_min_ = −0.41 e Å^−^^3^

### Special details

**Table:**

Experimental. The structure was solved by direct methods (Bruker, 2000) and successive difference Fourier syntheses.
Geometry. All e.s.d.'s (except the e.s.d. in the dihedral angle between two l.s. planes) are estimated using the full covariance matrix. The cell e.s.d.'s are taken into account individually in the estimation of e.s.d.'s in distances, angles and torsion angles; correlations between e.s.d.'s in cell parameters are only used when they are defined by crystal symmetry. An approximate (isotropic) treatment of cell e.s.d.'s is used for estimating e.s.d.'s involving l.s. planes.
Refinement. Refinement of *F*^2^ against ALL reflections. The weighted *R*-factor *wR* and goodness of fit *S* are based on *F*^2^, conventional *R*-factors *R* are based on *F*, with *F* set to zero for negative *F*^2^. The threshold expression of *F*^2^ > σ(*F*^2^) is used only for calculating *R*-factors(gt) *etc*. and is not relevant to the choice of reflections for refinement. *R*-factors based on *F*^2^ are statistically about twice as large as those based on *F*, and *R*- factors based on ALL data will be even larger.

### Fractional atomic coordinates and isotropic or equivalent isotropic displacement parameters (Å^2^)

**Table:**

	*x*	*y*	*z*	*U*_iso_*/*U*_eq_	
Ru1	0.25886 (3)	0.627135 (14)	0.52470 (3)	0.04287 (14)	
B1	0.7581 (7)	0.7190 (3)	0.5614 (8)	0.085 (2)	
B2	0.7624 (5)	0.4853 (3)	0.0278 (7)	0.0672 (16)	
C1	0.2611 (4)	0.5012 (2)	0.5649 (5)	0.0582 (12)	
H1	0.3145	0.5080	0.6341	0.070*	
C2	0.2343 (4)	0.4471 (2)	0.5328 (5)	0.0652 (13)	
H2	0.2692	0.4177	0.5788	0.078*	
C3	0.1540 (5)	0.4373 (2)	0.4303 (5)	0.0705 (14)	
H3	0.1351	0.4010	0.4060	0.085*	
C4	0.1029 (4)	0.4808 (2)	0.3653 (5)	0.0629 (12)	
H4	0.0477	0.4743	0.2974	0.075*	
C5	0.1331 (4)	0.53449 (18)	0.4002 (4)	0.0498 (10)	
C6	0.0783 (4)	0.5844 (2)	0.3409 (4)	0.0525 (11)	
C7	−0.0095 (4)	0.5832 (2)	0.2415 (4)	0.0639 (13)	
H7	−0.0311	0.5498	0.2024	0.077*	
C8	−0.0640 (4)	0.6321 (3)	0.2019 (5)	0.0716 (15)	
H8	−0.1226	0.6320	0.1353	0.086*	
C9	−0.0320 (5)	0.6811 (2)	0.2603 (5)	0.0725 (14)	
H9	−0.0706	0.7143	0.2368	0.087*	
C10	0.0588 (4)	0.6799 (2)	0.3549 (4)	0.0612 (12)	
H10	0.0826	0.7133	0.3928	0.073*	
C11	0.4260 (4)	0.5845 (2)	0.3099 (4)	0.0630 (12)	
H11	0.3897	0.5500	0.3263	0.076*	
C12	0.5170 (4)	0.5879 (2)	0.2173 (5)	0.0693 (14)	
H12	0.5413	0.5564	0.1719	0.083*	
C13	0.5713 (4)	0.6385 (2)	0.1930 (5)	0.0685 (14)	
H13	0.6339	0.6418	0.1312	0.082*	
C14	0.5321 (4)	0.6844 (2)	0.2612 (5)	0.0643 (13)	
H14	0.5685	0.7190	0.2461	0.077*	
C15	0.4388 (4)	0.67871 (19)	0.3518 (4)	0.0503 (10)	
C16	0.3859 (4)	0.72526 (18)	0.4252 (4)	0.0510 (10)	
C17	0.4096 (5)	0.7811 (2)	0.3994 (5)	0.0705 (14)	
H17	0.4663	0.7907	0.3353	0.085*	
C18	0.3501 (5)	0.8221 (2)	0.4676 (5)	0.0755 (15)	
H18	0.3659	0.8594	0.4503	0.091*	
C19	0.2667 (4)	0.8072 (2)	0.5620 (5)	0.0659 (13)	
H19	0.2246	0.8342	0.6093	0.079*	
C20	0.2469 (4)	0.75165 (19)	0.5852 (5)	0.0596 (12)	
H20	0.1923	0.7418	0.6509	0.071*	
C21	0.4890 (4)	0.6030 (2)	0.7128 (5)	0.0591 (12)	
C22	0.5932 (4)	0.5904 (3)	0.8000 (5)	0.0837 (17)	
H22A	0.5604	0.5836	0.8841	0.126*	
H22B	0.6505	0.6214	0.8028	0.126*	
H22C	0.6372	0.5579	0.7705	0.126*	
C23	0.0697 (4)	0.63709 (18)	0.7582 (4)	0.0531 (11)	
C24	−0.0175 (5)	0.6412 (2)	0.8654 (5)	0.0810 (16)	
H24A	−0.0613	0.6065	0.8756	0.122*	
H24B	−0.0777	0.6704	0.8490	0.122*	
H24C	0.0291	0.6496	0.9421	0.122*	
F1	0.7366 (4)	0.6846 (2)	0.4670 (4)	0.154 (2)	
F2	0.6838 (7)	0.7071 (2)	0.6605 (7)	0.262 (4)	
F3	0.7452 (4)	0.77248 (19)	0.5352 (4)	0.1427 (17)	
F4	0.8737 (5)	0.7071 (2)	0.6035 (6)	0.204 (3)	
F5	0.7715 (4)	0.5405 (2)	0.0135 (5)	0.168 (2)	
F6	0.8552 (5)	0.4701 (2)	0.1074 (6)	0.187 (2)	
F7	0.7700 (6)	0.4581 (3)	−0.0758 (6)	0.225 (3)	
F8	0.6515 (5)	0.4717 (2)	0.0777 (6)	0.185 (2)	
N1	0.2131 (3)	0.54494 (15)	0.5004 (3)	0.0477 (8)	
N2	0.1144 (3)	0.63331 (15)	0.3949 (3)	0.0479 (9)	
N3	0.3868 (3)	0.62815 (14)	0.3778 (3)	0.0472 (8)	
N4	0.3022 (3)	0.71070 (15)	0.5180 (3)	0.0481 (8)	
N5	0.4065 (3)	0.61253 (14)	0.6479 (3)	0.0482 (8)	
N6	0.1371 (3)	0.63333 (13)	0.6752 (3)	0.0474 (8)	

### Atomic displacement parameters (Å^2^)

**Table:**

	*U*^11^	*U*^22^	*U*^33^	*U*^12^	*U*^13^	*U*^23^
Ru1	0.0415 (2)	0.0481 (2)	0.0390 (2)	−0.00261 (15)	0.00032 (14)	0.00096 (15)
B1	0.082 (5)	0.071 (5)	0.103 (6)	0.019 (4)	−0.017 (4)	−0.032 (4)
B2	0.054 (3)	0.067 (4)	0.081 (4)	0.005 (3)	0.003 (3)	−0.004 (3)
C1	0.060 (3)	0.057 (3)	0.058 (3)	−0.006 (2)	−0.003 (2)	0.006 (2)
C2	0.072 (3)	0.059 (3)	0.065 (3)	0.001 (2)	0.006 (3)	0.010 (2)
C3	0.074 (3)	0.058 (3)	0.079 (4)	−0.013 (3)	0.010 (3)	−0.012 (3)
C4	0.061 (3)	0.066 (3)	0.062 (3)	−0.006 (2)	−0.002 (2)	−0.010 (3)
C5	0.047 (2)	0.058 (3)	0.044 (2)	−0.006 (2)	0.0064 (19)	−0.002 (2)
C6	0.044 (2)	0.069 (3)	0.044 (2)	−0.002 (2)	0.0036 (19)	−0.002 (2)
C7	0.049 (3)	0.093 (4)	0.050 (3)	−0.005 (3)	−0.001 (2)	−0.004 (3)
C8	0.050 (3)	0.115 (5)	0.049 (3)	0.007 (3)	−0.009 (2)	0.015 (3)
C9	0.064 (3)	0.093 (4)	0.060 (3)	0.012 (3)	−0.007 (2)	0.014 (3)
C10	0.060 (3)	0.065 (3)	0.059 (3)	0.005 (2)	0.001 (2)	0.007 (2)
C11	0.057 (3)	0.068 (3)	0.064 (3)	0.002 (2)	0.011 (2)	−0.005 (2)
C12	0.058 (3)	0.088 (4)	0.061 (3)	0.012 (3)	0.010 (2)	−0.009 (3)
C13	0.046 (3)	0.105 (4)	0.054 (3)	−0.001 (3)	0.010 (2)	0.004 (3)
C14	0.052 (3)	0.082 (4)	0.058 (3)	−0.014 (3)	0.003 (2)	0.005 (3)
C15	0.040 (2)	0.067 (3)	0.044 (2)	−0.005 (2)	−0.0024 (18)	0.004 (2)
C16	0.049 (2)	0.059 (3)	0.044 (2)	−0.010 (2)	−0.0080 (19)	0.003 (2)
C17	0.078 (3)	0.064 (3)	0.069 (3)	−0.019 (3)	0.008 (3)	0.011 (3)
C18	0.093 (4)	0.055 (3)	0.078 (4)	−0.020 (3)	−0.007 (3)	0.004 (3)
C19	0.071 (3)	0.058 (3)	0.069 (3)	0.000 (2)	−0.014 (3)	−0.004 (2)
C20	0.070 (3)	0.057 (3)	0.052 (3)	−0.007 (2)	0.003 (2)	0.000 (2)
C21	0.056 (3)	0.064 (3)	0.057 (3)	0.006 (2)	0.001 (2)	−0.003 (2)
C22	0.062 (3)	0.113 (5)	0.076 (4)	0.020 (3)	−0.013 (3)	−0.008 (3)
C23	0.056 (3)	0.060 (3)	0.043 (3)	−0.005 (2)	0.002 (2)	0.000 (2)
C24	0.074 (3)	0.110 (4)	0.060 (3)	−0.010 (3)	0.021 (3)	−0.014 (3)
F1	0.199 (5)	0.114 (4)	0.148 (4)	0.013 (3)	−0.070 (4)	−0.047 (3)
F2	0.362 (9)	0.134 (4)	0.290 (8)	0.038 (5)	0.217 (8)	0.024 (5)
F3	0.185 (5)	0.087 (3)	0.156 (4)	0.012 (3)	0.004 (3)	0.004 (2)
F4	0.183 (5)	0.177 (5)	0.251 (6)	0.055 (4)	−0.123 (5)	−0.088 (4)
F5	0.161 (4)	0.103 (4)	0.240 (6)	−0.021 (3)	−0.084 (4)	0.052 (3)
F6	0.187 (5)	0.150 (4)	0.222 (6)	−0.007 (3)	−0.107 (5)	0.065 (4)
F7	0.207 (6)	0.311 (9)	0.158 (5)	−0.039 (5)	0.047 (4)	−0.139 (6)
F8	0.138 (4)	0.132 (4)	0.285 (7)	−0.007 (3)	0.095 (4)	−0.011 (4)
N1	0.0453 (18)	0.053 (2)	0.045 (2)	−0.0040 (16)	0.0028 (16)	0.0007 (16)
N2	0.0408 (18)	0.062 (2)	0.041 (2)	−0.0011 (16)	−0.0002 (15)	0.0067 (17)
N3	0.0438 (18)	0.057 (2)	0.041 (2)	−0.0028 (17)	−0.0023 (15)	−0.0027 (16)
N4	0.0497 (19)	0.052 (2)	0.043 (2)	−0.0035 (17)	−0.0043 (16)	0.0003 (16)
N5	0.049 (2)	0.052 (2)	0.044 (2)	0.0002 (17)	0.0007 (17)	0.0009 (16)
N6	0.047 (2)	0.051 (2)	0.045 (2)	−0.0016 (16)	−0.0025 (17)	0.0013 (16)

### Geometric parameters (Å, °)

**Table:**

Ru1—N6	2.042 (4)	C10—N2	1.330 (5)
Ru1—N2	2.043 (3)	C10—H10	0.9300
Ru1—N1	2.049 (4)	C11—N3	1.333 (5)
Ru1—N5	2.049 (4)	C11—C12	1.369 (6)
Ru1—N3	2.051 (3)	C11—H11	0.9300
Ru1—N4	2.060 (4)	C12—C13	1.369 (7)
B1—F1	1.307 (8)	C12—H12	0.9300
B1—F3	1.320 (8)	C13—C14	1.378 (7)
B1—F4	1.328 (7)	C13—H13	0.9300
B1—F2	1.334 (9)	C14—C15	1.377 (6)
B2—F7	1.268 (7)	C14—H14	0.9300
B2—F8	1.326 (7)	C15—N3	1.361 (5)
B2—F6	1.335 (7)	C15—C16	1.468 (6)
B2—F5	1.337 (8)	C16—N4	1.361 (5)
C1—N1	1.346 (6)	C16—C17	1.391 (6)
C1—C2	1.372 (7)	C17—C18	1.371 (7)
C1—H1	0.9300	C17—H17	0.9300
C2—C3	1.384 (7)	C18—C19	1.373 (7)
C2—H2	0.9300	C18—H18	0.9300
C3—C4	1.356 (6)	C19—C20	1.372 (6)
C3—H3	0.9300	C19—H19	0.9300
C4—C5	1.378 (6)	C20—N4	1.344 (6)
C4—H4	0.9300	C20—H20	0.9300
C5—N1	1.366 (5)	C21—N5	1.126 (5)
C5—C6	1.467 (6)	C21—C22	1.458 (6)
C6—N2	1.358 (5)	C22—H22A	0.9600
C6—C7	1.390 (6)	C22—H22B	0.9600
C7—C8	1.371 (7)	C22—H22C	0.9600
C7—H7	0.9300	C23—N6	1.129 (5)
C8—C9	1.366 (7)	C23—C24	1.457 (6)
C8—H8	0.9300	C24—H24A	0.9600
C9—C10	1.375 (6)	C24—H24B	0.9600
C9—H9	0.9300	C24—H24C	0.9600
			
N6—Ru1—N2	92.02 (13)	N3—C11—H11	118.3
N6—Ru1—N1	90.96 (13)	C12—C11—H11	118.3
N2—Ru1—N1	79.18 (14)	C13—C12—C11	118.7 (5)
N6—Ru1—N5	90.50 (13)	C13—C12—H12	120.7
N2—Ru1—N5	173.93 (14)	C11—C12—H12	120.7
N1—Ru1—N5	95.25 (13)	C12—C13—C14	119.2 (4)
N6—Ru1—N3	174.73 (13)	C12—C13—H13	120.4
N2—Ru1—N3	89.67 (13)	C14—C13—H13	120.4
N1—Ru1—N3	94.26 (13)	C15—C14—C13	119.6 (5)
N5—Ru1—N3	88.31 (13)	C15—C14—H14	120.2
N6—Ru1—N4	95.53 (13)	C13—C14—H14	120.2
N2—Ru1—N4	94.13 (13)	N3—C15—C14	121.1 (4)
N1—Ru1—N4	170.86 (13)	N3—C15—C16	114.9 (4)
N5—Ru1—N4	91.12 (13)	C14—C15—C16	124.0 (4)
N3—Ru1—N4	79.36 (14)	N4—C16—C17	120.3 (4)
F1—B1—F3	116.2 (7)	N4—C16—C15	115.3 (4)
F1—B1—F4	105.7 (6)	C17—C16—C15	124.3 (4)
F3—B1—F4	111.9 (7)	C18—C17—C16	120.5 (5)
F1—B1—F2	110.5 (7)	C18—C17—H17	119.7
F3—B1—F2	108.1 (6)	C16—C17—H17	119.7
F4—B1—F2	103.9 (8)	C17—C18—C19	119.0 (5)
F7—B2—F8	105.6 (6)	C17—C18—H18	120.5
F7—B2—F6	110.1 (6)	C19—C18—H18	120.5
F8—B2—F6	109.6 (6)	C20—C19—C18	118.6 (5)
F7—B2—F5	114.3 (7)	C20—C19—H19	120.7
F8—B2—F5	110.6 (5)	C18—C19—H19	120.7
F6—B2—F5	106.7 (5)	N4—C20—C19	123.5 (5)
N1—C1—C2	122.7 (4)	N4—C20—H20	118.2
N1—C1—H1	118.7	C19—C20—H20	118.2
C2—C1—H1	118.7	N5—C21—C22	178.3 (5)
C1—C2—C3	118.4 (5)	C21—C22—H22A	109.5
C1—C2—H2	120.8	C21—C22—H22B	109.5
C3—C2—H2	120.8	H22A—C22—H22B	109.5
C4—C3—C2	119.9 (5)	C21—C22—H22C	109.5
C4—C3—H3	120.0	H22A—C22—H22C	109.5
C2—C3—H3	120.0	H22B—C22—H22C	109.5
C3—C4—C5	119.8 (5)	N6—C23—C24	179.3 (5)
C3—C4—H4	120.1	C23—C24—H24A	109.5
C5—C4—H4	120.1	C23—C24—H24B	109.5
N1—C5—C4	121.1 (4)	H24A—C24—H24B	109.5
N1—C5—C6	114.5 (4)	C23—C24—H24C	109.5
C4—C5—C6	124.2 (4)	H24A—C24—H24C	109.5
N2—C6—C7	120.9 (4)	H24B—C24—H24C	109.5
N2—C6—C5	114.9 (4)	C1—N1—C5	118.1 (4)
C7—C6—C5	124.1 (4)	C1—N1—Ru1	127.0 (3)
C8—C7—C6	119.1 (5)	C5—N1—Ru1	114.7 (3)
C8—C7—H7	120.4	C10—N2—C6	118.3 (4)
C6—C7—H7	120.4	C10—N2—Ru1	126.7 (3)
C9—C8—C7	119.9 (5)	C6—N2—Ru1	114.9 (3)
C9—C8—H8	120.0	C11—N3—C15	118.0 (4)
C7—C8—H8	120.0	C11—N3—Ru1	126.7 (3)
C8—C9—C10	118.3 (5)	C15—N3—Ru1	115.3 (3)
C8—C9—H9	120.8	C20—N4—C16	118.0 (4)
C10—C9—H9	120.8	C20—N4—Ru1	126.7 (3)
N2—C10—C9	123.3 (5)	C16—N4—Ru1	114.8 (3)
N2—C10—H10	118.3	C21—N5—Ru1	177.5 (4)
C9—C10—H10	118.3	C23—N6—Ru1	179.6 (4)
N3—C11—C12	123.4 (5)		

### Hydrogen-bond geometry (Å, °)

**Table:**

*D*—H···*A*	*D*—H	H···*A*	*D*···*A*	*D*—H···*A*
C2—H2···F1^i^	0.93	2.50	3.179 (7)	130
C7—H7···F6^ii^	0.93	2.47	3.373 (7)	165
C9—H9···F4^iii^	0.93	2.42	3.299 (7)	158
C12—H12···F8	0.93	2.54	3.459 (7)	167
C14—H14···F2^iv^	0.93	2.33	3.238 (8)	164

Symmetry codes: (i) −*x*+1, −*y*+1, −*z*+1; (ii) *x*−1, *y*, *z*; (iii) *x*−1, −*y*+3/2, *z*−1/2; (iv) *x*, −*y*+3/2, *z*−1/2.

## References

[bb1] Bruker (2000). *SMART*, *SAINT* and *SADABS* Bruker AXS Inc., Madison, Wisconsin, USA.

[bb2] Chattopadhyay, S. K., Mitra, K., Biswas, S., Naskar, S., Mishra, D., Adhikary, B., Harrison, R. G. & Cannon, J. F. (2004). *Transition Met. Chem.* **29**, 1–6.

[bb3] Cordes, A. W., Durham, B., Pennington, W. T., Kuntz, B. & Allen, L. (1992). *J. Crystallogr. Spectrosc. Res.* **22**, 699–704.

[bb4] Heeg, M. J., Kroener, R. & Deutsch, E. (1985). *Acta Cryst.* C**41**, 684–686.

[bb5] Sheldrick, G. M. (2008). *Acta Cryst.* A**64**, 112–122. 10.1107/S0108767307043930 18156677

[bb6] Xu, F. & Huang, W. (2007). *Acta Cryst.* E**63**, m2114.

